# Struggling for breath in Sherbrooke - 1st Symposium on “One mitochondrion, many diseases” in Sherbrooke, Québec, Canada, March 11th, 2015

**DOI:** 10.15698/mic2015.06.207

**Published:** 2015-05-20

**Authors:** Ralf J. Braun, Verónica I. Dumit, Cécile Monpays, Xavier Roucou, Daniel Serrano, Julie St-Pierre, Paula J. Waters, Ian Bates, Denis Gris

**Affiliations:** 1 Institute of Cell Biology, University of Bayreuth, 95440 Bayreuth, Germany.; 2 ZBSA Center for Biological Systems Analysis, Core Facility Proteomics, University of Freiburg, Freiburg, Germany.; 3 Department of Dermatology, Medical Center - University of Freiburg, Freiburg, Germany.; 4 Department of Pharmacology and Physiology, University of Sherbrooke Hospital Centre (CHUS), Sherbrooke, QC, Canada.; 5 Department of Biochemistry, Faculty of Medicine and Health Sciences, University of Sherbrooke, Sherbrooke, QC, Canada.; 6 Immunology Division, Department of Pediatrics, Faculty of Medicine and Health Sciences, University of Sherbrooke, Sherbrooke, QC, Canada.; 7 Goodmann Cancer Research Centre & Department of Biochemistry, McGill University, Montréal, QC, Canada.; 8 Medical Genetics Service, Department of Pediatrics, University of Sherbrooke Hospital Centre (CHUS), Sherbrooke, QC, Canada.; 9 Carl Zeiss Canada MicroImaging, Toronto, ON, Canada.; 10 Program of Immunology, Department of Pediatrics, CR-CHUS, Faculty of Medicine and Health Sciences, University of Sherbrooke, Sherbrooke, QC, Canada.

**Keywords:** mitochondria, neurodegeneration, cancer, mitochondrial disorders, mitochondrial dynamics, metabolic adaption, oxidative stress, programmed cell death, apoptosis, necrosis, inflammation, neuronal cell culture, transgenic mouse models, Saccharomyces cerevisiae

## INTRODUCTION

On March 11^th^ 2015, when the never-ending bitterly cold Canadian winter made a short break, 100 scientists from Québec and Germany came together in Sherbrooke in southern Québec to attend the 1^st^ symposium on “One mitochondrion, many diseases”. Under the impression of the beauty of the frozen local rivers Saint-François and Magog, the researchers presented and discussed their recent data on the pivotal roles of mitochondria in various diseases, including neurodegeneration, cancer, schizophrenia, respiratory chain, and inflammatory disorders. In this way, the participants and attendees of the symposium got a broad overview of common and distinct mechanisms of mitochondrial (dys)functions underlying various diseases.

## MITOCHONDRIAL-DERIVED VESICLES IN MITOCHONDRIAL QUALITY CONTROL AND NEURODEGENERATION

**Figure 1:**
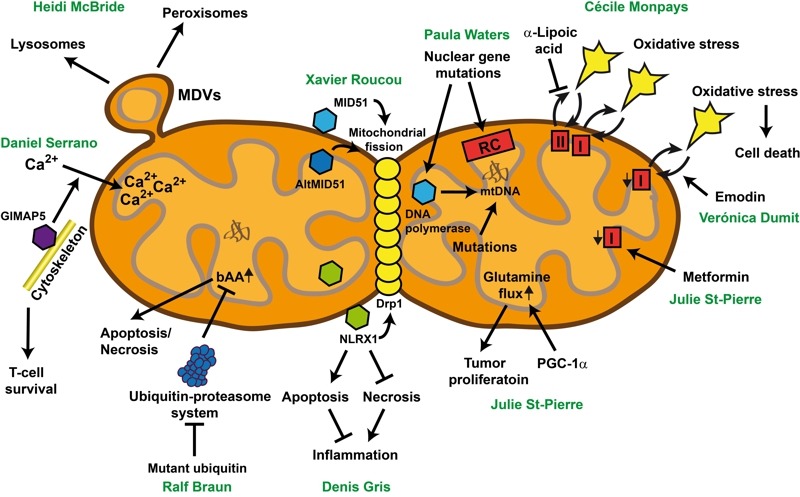
FIGURE 1: One mitochondrion, various disease-relevant pathways. For details see text. bAA: basic amino acids, MDV: mitochondrial-derived vesicles.

**Heidi McBride** (McGill University, Montréal, QC, Canada) was the first keynote speaker of the symposium. She presented mitochondria as very dynamic organelles, which tightly interact with an extended network of intracellular organelles, including the ER, lysosomes, early and late endosomes, and peroxisomes. Notably, mitochondria are able to bud vesicles. These mitochondrial-derived vesicles (MDVs) play important roles in maintaining mitochondrial quality control, and potentially contribute to peroxisomal function (Figure 1).

One type of MDVs is targeted to the lysosomes. Here, MDVs are stimulated upon oxidative stress, and this machinery runs in parallel to mitophagy. MDVs targeted to lysosomes and mitophagy share some components, including the mitochondrial kinase Pink1 and the E3 ligase Parkin, which both are upon mutation causative for Parkinson’s disease 1. Heidi McBride proposed that MDVs targeted to lysosomes and mitophagy are two independent mitochondrial quality control mechanisms. The first one enables the removal of parts of dysfunctional mitochondria upon mild oxidative stress, whereas the latter removes whole mitochondrial entities, which are damaged upon strong oxidative stress. MDVs are formed faster than mitophagy can proceed, suggesting that the formation of MDVs is the primary quality control mechanism. Only when this mechanism is overwhelmed, mitophagy is induced to prevent cells from the accumulation of damaged mitochondria.

In yeast cells peroxisomes are formed *de novo* from the ER, as evidenced by the first appearance of the peroxisomal membrane protein Pex3 in the ER before reaching the peroxisomes. In contrast, in mammalian cells the default localization of Pex3 appears to be the mitochondrial outer membrane, suggesting that mitochondria contribute to the formation of peroxisomes. Previously, Heidi McBride resolved the cellular machinery of MDV formation towards the peroxisomes, which depends on the mitochondrial-anchored protein ligase MAPL 2. Now, she aims to understand whether the MDV transport to peroxisomes is pivotal for peroxisome formation and function.

## STRUCTURAL AND METABOLIC ADAPTIONS IN MITOCHONDRION-DEPENDENT CELL DEATH IN NEURODEGENERATION

**Denis Gris** (University of Sherbrooke, Sherbrooke, QC, Canada) analyzes the role of proteins involved in the innate immune response on the survival of neurons. He is working on members of the nucleotide-binding domain leucine-rich repeat-containing protein (NLR) family, which act as molecular switches that redirect multiple signaling pathways. He presented his work on NLRX1, the only member of the NLR family with mitochondrial localization sequence (Figure 1). Knock-down of NLRX1 in neuronal cells triggers necrotic cell death 3. In contrast, increased levels of NLRX1 protect cells from the toxicity of the toxin rotenone, which affects complex I activity of the mitochondrial respiratory chain 3. Depletion of NLRX1 shifts the ratio of apoptosis to necrosis towards necrosis upon rotenone treatment 3. Since necrotic cell death of neurons, in contrast to apoptotic cell death, leads to an inflammatory response, NLRX1 could be a molecular switch controlling both neuronal survival and inflammatory signaling. NLRX1 activities have important effects on the structure of both the mitochondrial network, and the inner-mitochondrial organization. NLRX1 associates with the mitochondrial fission protein Drp1, triggers its phosphorylation and activation, culminating in increased mitochondrial fission 3. This could lead to the observed increased mitochondrial mass in cells with high levels of NLRX1. In parallel, the number of cristae in mitochondria is markedly reduced. Thus, NLRX1 is involved in controlling mitochondrial structure and mitochondrion-dependent apoptosis and necrosis.

Data obtained from a yeast model expressing Alzheimer’s disease-associated mutant ubiquitin were presented from **Ralf Braun** (University of Bayreuth, Bayreuth, Germany). He demonstrated that accumulation of mutant ubiquitin impairs the ubiquitin-proteasome system (UPS), leads to pivotal mitochondrial impairment, culminating in apoptotic and necrotic cell death 4. Surprisingly, mutant ubiquitin accumulation leads to the enrichment of enzymes in or at mitochondria, which are crucial for the production of the basic amino acids arginine, ornithine, and lysine (Figure 1). Consistently, these basic amino acids accumulate in cells with high levels of mutant ubiquitin and are important for the execution of mitochondrion-mediated cell death 4. Promoting the mitochondrion-associated branch of the UPS was sufficient to reduce the cellular levels of basic amino acids, to protect mitochondria and to prevent cell death in the presence of high levels of mutant ubiquitin 4. These data indicate a pivotal role of UPS (dys)function in controlling metabolic activities in mitochondria, which could be important for the progression of neurodegenerative disorders, in which UPS dysfunction and mitochondrial damage are common hallmarks.

## MITOCHONDRIA AND METABOLIC ADAPTATION IN CANCER

Deregulation of cellular energetics is a hallmark of cancer. The second keynote speaker of the symposium, **Julie St-Pierre** (McGill University, Montréal, QC, Canada) demonstrated that mitochondrial metabolism is altered during the metabolic adaptations in cancer cells. She established unique methods enabling the metabolite profiling of cancer and control cells, including the targeted profile of all intermediates in the citric acid cycle 5, and the execution of stable isotope tracer analyses in isolated mitochondria 6. Applying these methods, she analyzed the role of the peroxisome proliferator-activated receptor γ coactivator 1α (PGC-1α) in cancer, as well as the impact of the diabetic drug metformin on cancer cell metabolism. Intense efforts are currently underway to investigate whether metformin could have therapeutic benefit in cancer given that some studies had reported decreased cancer risk in patients taking metformin.

PGC-1α regulates the metabolism of multiple tissues by promoting mitochondrial biogenesis, respiration, and reactive oxygen species detoxifying capacity. Several reports have shown that the expression of PGC-1α is decreased in breast cancer patients compared with normal tissues. However, PGC-1α expression is highest in HER2+ and triple negative breast cancers, subtypes that have the poorest prognosis 7. Julie St-Pierre’s work uncovered that PGC-1α promotes the growth of ErbB2/Neu-initiated mammary tumors by increasing their nutrient availability 8. Glutamine cooperates with glucose in supporting tumor metabolism, and Julie St-Pierre showed that PGC-1α increases glutamine uptake and promotes the expression of the glutamine metabolism genes, thereby augmenting the glutamine flow (both forward and reverse) through the citric acid cycle 9 (Figure 1). The clinical relevance of her work is illustrated by the fact that in breast cancer patients, PGC-1α expression is positively correlated with that of the enzymes of the glutamine pathway, and that high expression of this pathway is associated with reduced survival 9.

Next, Julie St-Pierre investigated the metabolic target of metformin. Despite its common usage, the target of metformin has remained controversial. Using respirometry and metabolite profiling, she showed that cancer cells treated with metformin demonstrate increased glycolysis and impaired respiratory activities 10. Importantly, she revealed that metformin directly acts on mitochondria by inhibiting complex I- but not complex II-dependent respiration, causing alterations in citric acid cycle functions (Figure 1). Cancer cells were more energetically stressed by metformin exposure than non-transformed controls 10. Overall, Julie St-Pierre work shows that cancer cells display unique metabolic adaptations that may provide opportunities for therapeutic interventions.

**Verónica Dumit** (University of Freiburg, Freiburg, Germany) presented her data using leaf extracts prepared from the aloe plant to trigger cell death in cancer cells but not in healthy control fibroblasts. She applied a quantitative proteomic analysis using stable isotope labeling in cell culture (SILAC) to determine protein alterations in cancer and healthy cells upon aloe extract treatment, and observed the downregulation of proteins involved in DNA replication and mitochondrial energy metabolism. Treatment with emodin, an anthraquinone component of aloe, resulted in similar results as compared to total aloe extracts. It leads to the downregulation of mitochondrial complex I subunits in cancer cells, and triggers mitochondrial fragmentation and ballooning (Figure 1). Emodin shifts isolated yeast mitochondria towards uncoupled respiration, affects the mitochondrial membrane potential, which then leads to impaired import of proteins into the mitochondrial matrix. Yeast cells adapted to fermentation are much more vulnerable to emodin treatment than yeast cells with high respiratory capacity. This effect might be comparable to cancer cells which prefer fermentation in contrast to control cells which demonstrate higher levels of respiratory activities. Both fermenting yeast and cancer cells can be protected from the detrimental effects of emodin by using the antioxidant *N*-acetyl-*L*-cysteine (NAC), demonstrating an important role of ROS in the execution of cell death. In summary, emodin triggers death of cells with an inefficient mitochondrial respiratory chain, such as cancer cells.

## MITOCHONDRIAL ACTIVITIES IN SCHIZOPHRENIA

**Cécile Monpays** (University of Sherbrooke, Sherbrooke, QC, Canada) determined the mitochondrial activities in a mice juvenile two-hit model of schizophrenia. Schizophrenia is a chronic mental illness which affects 1% of general population and is characterized by different clinical symptoms with three core features, positive (e.g. hallucination), negative (e.g. lack of motivation) and cognitive (e.g. attention deficit symptoms). These clinical symptoms are related to different neurochemical perturbations, among which are oxidative stress and energetic status disturbances. In schizophrenia, the imbalance between production of reactive oxygen species and activity of antioxidant enzymes points towards mitochondrial dysfunction. The colleagues of Cécile Monpays have developed a juvenile murine two-hit model of schizophrenia based on the combination of two environmental risk factors (gestational inflammation induced by poly IC followed by juvenile restraint stress) to get a better understanding of the disease onset 11. They reported relevant behaviors and neurochemical disturbances, including oxidative stress, thus providing preliminary validation of this model 11 (Figure 1). These deficits were reversed by the antioxidant α-lipoic acid, pointing to the central role of oxidative abnormalities. Cécile Monpays and colleagues examined the mitochondrial function in the juvenile murine two-hit model of schizophrenia within two relevant regions (prefrontal cortex and striatum) associated with schizophrenia. They observed an increase of complex I-induced respiratory activity in the prefrontal cortex and striatum in both sexes but an increase of complex II activity only in males. α-lipoic acid treatment only prevented the increase in complex II-induced respiration in males but had no effect on complex I-induced activity (Figure 1). In the next steps Cécile Monpays and colleagues will measure the expression of the respiratory chain complexes and fission/fusion proteins, and will determine the leak respiration and its potential relationship with the well-described glutamatergic abnormalities in schizophrenia.

## MITOCHONDRIAL RESPIRATORY CHAIN DISORDERS

Defects in the structural and functional integrity of the mitochondrial respiratory chain (RC) can lead to significant mitochondrial disorders, with a wide variety of phenotypes. **Paula Waters** (University of Sherbrooke, Sherbrooke, QC, Canada) gave an overview of clinical, biochemical and genetic aspects of mitochondrial RC disorders, dividing these into three broad classes (Figure 1) and presenting selected case histories illustrative of each.

The first class involves mutations in nuclear genes encoding protein subunits of the mitochondrial RC or encoding accessory proteins essential for assembly and stability of RC complexes. She presented the history of a family with children suffering from Leigh syndrome, caused by mutations in the *SURF1* gene which encodes the protein surfeit locus protein 1 (Surf1). This protein localizes to the mitochondrial inner membrane and is involved in the biogenesis of the cytochrome *c* oxidase complex (RC complex IV).

The second class of mitochondrial RC disorders involves mutations in the nuclear genome which, in turn, affect the mitochondrial genome (mtDNA). Dr. Waters presented the history of an infant with Alpers syndrome, due to mutations in the nuclear gene *POLG1*. This gene encodes a subunit of the mitochondrial DNA polymerase γ, an enzyme with a pivotal role in mtDNA replication. The mutations affected mtDNA integrity, thereby impairing mitochondrial RC activities.

The third class of mitochondrial RC disorders involves mutations in the mitochondrial genome. The example presented was the history of a child with NARP (Neurogenic muscle weakness, Ataxia and Retinitis Pigmentosa) caused by the m.8993T>C mutation, affecting a subunit of the mitochondrial ATPase complex (RC complex V). Complex issues facing families with pathogenic mtDNA mutations were highlighted. Mutations in the mtDNA demonstrate matrilineal transmission (mitochondria are only transmitted by the mother and not by the father) and therefore do not follow Mendelian inheritance. Heteroplasmy, i.e. a mixed population of mitochondria with wild-type and mutated mtDNA, can lead to incomplete penetrance, variable expressivity, and pleiotropy in this class of mtDNA disorders. Novel assisted reproductive technologies, designed to uncouple the inheritance of nuclear and mtDNA and thus to minimize the risk of transmission of mtDNA disorders, have recently been developed. These techniques, their potential clinical applications and wider implications, are currently the subject of active public discussion in many countries 12.

## MITOCHONDRIA IN T CELL SURVIVAL

The GTPase of the immune-associated nucleotide-binding protein 5 (GIMAP5) plays a pro-survival function in T-lymphocytes. Deletion of GIMAP5 results in spontaneous apoptosis in mature T-lymphocytes in rats, and impairs the entry of Ca^2+^ ions via plasma membrane channels. **Daniel Serrano** (University of Sherbrooke, Sherbrooke, QC, Canada) presented data demonstrating that this is due to the inability of mitochondria in GIMAP5-deficient T-cells to sequester Ca^2+^ 13 (Figure 1). GIMAP5 partially co-localizes with kinesin, the motor protein crucial for the anterograde transport along the microtubule cytoskeleton. Consistently, microtubules play an important role in mitochondrial Ca^2+^ sequestration. GIMAP5 increases the capacity of mitochondria for Ca^2+^, highlighting the tight interconnection among the microtubule cytoskeleton and mitochondria in promoting survival of naïve T cells.

## CONSIDERATION OF THE ALTERNATIVE PROTEOME FOR DISEASE AND MITOCHONDRIAL DYNAMICS

Mature mRNA contains unconventional open reading frames (AltORFs) located in the untranslated regions or overlapping the reference ORFs (RefORFs) in non-canonical +2 and +3 reading frames 14. **Xavier Roucou** (University of Sherbrooke, Sherbrooke, QC, Canada) generated proteome databases including both the reference and the predicted alternative ORFs for different species, including humans and yeast. Based on these extended databases, he was able to identify numerous hitherto undetectable and unknown small proteins encoded by AltORFs 14. Among them he identified AltMID51 encoded by an AltORF in the mRNA encoding the RefORF of the mitochondrial dynamics protein of 51 kDa (MID51) (Figure 1). AltMID51 turned out to be a LYR protein family member, which are components of mitochondrial protein complexes. Indeed, AltMID51 co-localized with mitochondria in cell culture, and homodimerized in mitochondrial foci in a manner depending on the LYR domain. The ORFs encoding AltMID51 and MID51 are evolutionary tightly associated, and overexpression of both proteins triggered mitochondrial fragmentation. Further examination will elucidate whether AltMID51 and MID51 are involved in similar cellular pathways, which has already been shown for other proteins, including disease-associated proteins, encoded by the AltORF and the respective RefORF.

## CHALLENGES AND OPPORTUNITIES OF SUPER-RESOLUTION IMAGING IN MITOCHONDRIAL RESEARCH

**Ian Bates** (Carl Zeiss Canada) briefly introduced super-resolution fluorescence microscopy. He reviewed existing super-resolution methods and discussed the advantages and disadvantages of existing techniques based on the particular scientific question. In particular live cell imaging is a challenge for super resolution imaging, since traditionally images either take a significant amount of time to acquire and/or require high laser powers, both conditions are not conducive to live cell imaging. Techniques which can both improve sensitivity and resolution are the ideal solution for maintaining cell viability yet at the same time reveal new biological information about dynamic processes seen in mitochondrial research in particular. This is the new frontier in super-resolution microscopy, and the power of these methods will bring new insights into the role mitochondria plays in various human disorders.

## CONCLUDING REMARKS

Mitochondria are fascinating highly dynamic organelles which play important roles in many cellular processes, including ATP production via respiratory chain activities, amino acid metabolism, and Ca^2+^ homeostasis. Therefore, it is hardly surprising that mitochondrial (dys)functions play important roles in various human disorders. In this symposium, the role of mitochondria was described in human patients as well as in mammalian cell culture, yeast, and transgenic mouse models for different diseases. It was fascinating to see how basic research resulted in the identification of mitochondrial-derived vesicular transport processes, which turned out to be relevant for neurodegeneration. Alterations in mitochondrion-localized metabolic processes and the activities of distinct complexes of the respiratory chain were discovered to be important in both cancer and neurodegeneration. Structural alteration of mitochondria and the switch of apoptosis to necrosis is relevant for inflammatory and neurodegenerative processes. In our next symposium, which will take place in Germany in 2016, we will continue our successful concept of bringing mitochondrial researchers together doing basic or clinical research, or working on diverse human disorders in different model systems. We trust that this was and will be a fruitful approach to think about common and distinct mechanisms of mitochondria in different pathophysiological conditions.

## References

[1] McLelland GL, Soubannier V, Chen CX, McBride HM, Fon EA (2014). Parkin and PINK1 function in a vesicular trafficking pathway regulating mitochondrial quality control.. EMBO J.

[2] Braschi E, Goyon V, Zunino R, Mohanty A, Xu L, McBride HM (2010). Vps35 mediates vesicle transport between the mitochondria and peroxisomes.. Curr Biol.

[3] Imbeault E, Mahvelati TM, Braun R, Gris P, Gris D (2014). Nlrx1 regulates neuronal cell death.. Mol Brain.

[4] Braun RJ, Sommer C, Leibiger C, Gentier RJ, Dumit VI, Paduch K, Eisenberg T, Habernig L, Trausinger G, Magnes C, Pieber T, Sinner F, Dengjel J, van Leeuwen FW, Kroemer G, Madeo F (2015). Accumulation of Basic Amino Acids at Mitochondria Dictates the Cytotoxicity of Aberrant Ubiquitin.. Cell Rep.

[5] Mamer O, Gravel SP, Choiniere L, Chenard V, St-Pierre J, Avizonis D (2013). The complete targeted profile of the organic acid intermediates of the citric acid cycle using a single stable isotope dilution analysis, sodium borodeuteride reduction and selected ion monitoring GC/MS.. Metabolomics.

[6] Gravel SP, Andrzejewski S, Avizonis D, St-Pierre J (2014). Stable isotope tracer analysis in isolated mitochondria from mammalian systems.. Metabolites.

[7] Deblois G, St-Pierre J, Giguere V (2013). The PGC-1/ERR signaling axis in cancer.. Oncogene.

[8] Klimcakova E, Chenard V, McGuirk S, Germain D, Avizonis D, Muller WJ, St-Pierre J (2012). PGC-1alpha promotes the growth of ErbB2/Neu-induced mammary tumors by regulating nutrient supply.. Cancer Res.

[9] McGuirk S, Gravel SP, Deblois G, Papadopoli DJ, Faubert B, Wegner A, Hiller K, Avizonis D, Akavia UD, Jones RG, Giguere V, St-Pierre J (2013). PGC-1alpha supports glutamine metabolism in breast cancer.. Cancer Metab.

[10] Andrzejewski S, Gravel SP, Pollak M, St-Pierre J (2014). Metformin directly acts on mitochondria to alter cellular bioenergetics.. Cancer Metab.

[11] Deslauriers J, Racine W, Sarret P, Grignon S (2014). Preventive effect of alpha-lipoic acid on prepulse inhibition deficits in a juvenile two-hit model of schizophrenia.. Neuroscience.

[12] Richardson J, Irving L, Hyslop LA, Choudhary M, Murdoch A, Turnbull DM, Herbert M (2015). Concise reviews: Assisted reproductive technologies to prevent transmission of mitochondrial DNA disease.. Stem cells.

[13] Chen XL, Serrano D, Mayhue M, Wieden HJ, Stankova J, Boulay G, Ilangumaran S, Ramanathan S (2013). GTPase of the immune-associated nucleotide-binding protein 5 (GIMAP5) regulates calcium influx in T-lymphocytes by promoting mitochondrial calcium accumulation.. Biochem J.

[14] Vanderperre B, Lucier JF, Bissonnette C, Motard J, Tremblay G, Vanderperre S, Wisztorski M, Salzet M, Boisvert FM, Roucou X (2013). Direct detection of alternative open reading frames translation products in human significantly expands the proteome.. PLoS One.

